# Successful glenoid defect management with antibiotic-eluting bone graft substitute following baseplate removal in two-stage revision reverse shoulder arthroplasty: a case report and surgical considerations

**DOI:** 10.1016/j.xrrt.2026.100866

**Published:** 2026-09-25

**Authors:** Elise Beijer, Michel P.J. van den Bekerom, Robert J. Derksen

**Affiliations:** a Department of Orthopaedic Surgery, Shoulder and Elbow Unit, Joint Research, OLVG, Amsterdam, The Netherlands; b Department of Surgery, Section Trauma Surgery, Amsterdam University Medical Centers, VU University Amsterdam, The Netherlands; c Department of Anesthesiology, Amsterdam University Medical Centers, VU University Amsterdam, The Netherlands; d Laboratory of Experimental Intensive Care and Anesthesiology (LEICA), Amsterdam University Medical Centers, University of Amsterdam, The Netherlands; e Faculty of Behavioural and Movement Sciences, Vrije Universiteit Amsterdam, Amsterdam, The Netherlands; f Department of Trauma Surgery, Zaandam Medical Center, Zaandam, The Netherlands

**Figure:**
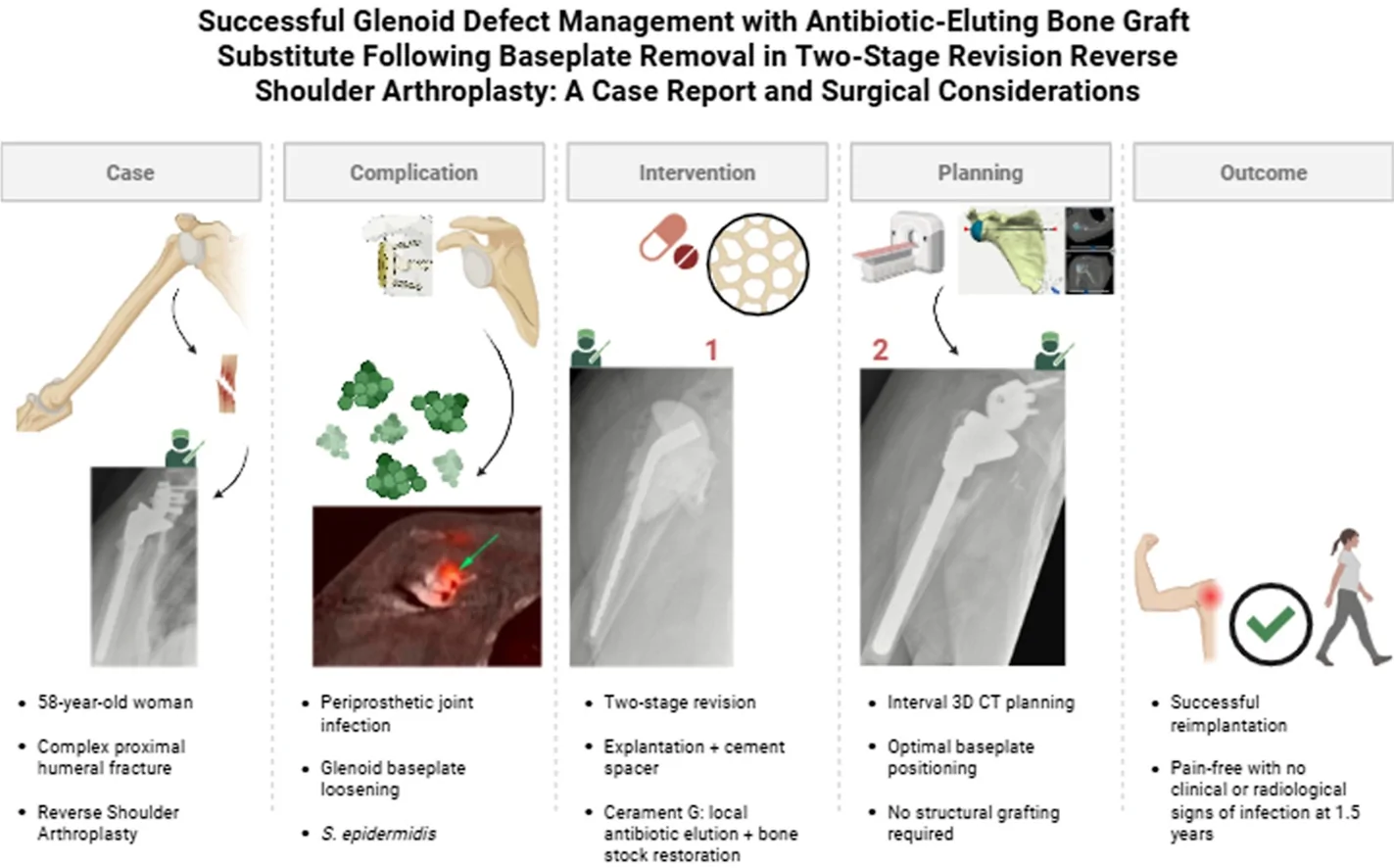

## Introduction

Reverse shoulder arthroplasty (rTSA) is an increasingly adopted solution across a broadening range of indications,^7^^,^^19^^,^^35^^,^^38^ and with rising procedure volumes, a corresponding rise in revision burden is anticipated in the coming years.^23^^,^^25^^,^^26^ Revision of the glenoid baseplate, indicated for aseptic loosening, periprosthetic joint infection (PJI), conversion from anatomic to reverse arthroplasty, or correction of baseplate malposition, presents a considerable surgical challenge demanding meticulous pre-operative planning and strategy, largely dictated by the extent of residual bone stock and the presence or suspicion of infection. Several classification systems provide a useful framework for current surgical decision-making.^5^^,^^18^ The Seebauer classification focusses more on the morphology of the defect and distinguishes between centric and eccentric defects: centric defects (C1-C4) range from shallow contained cavitary lesions to destructive vault involvement, while eccentric defects (E1-E4) are further characterized by size and anatomical location. The Göhlke classification focuses on surgical strategy and describes that mild defects (type 1) are amenable to impaction bone grafting combined with reverse baseplate implantation; moderate defects (type 2) usually require additional biological rTSA (allograft or autograft impaction) prior to baseplate fixation; and severe defects with anterior or posterior wall insufficiency (type 3) necessitate structural biological rTSA, usually with graft fixation and the placement of multiple screws. The choice between allograft and autograft carries important trade-offs. In the primary setting, autograft can be harvested from the resected humeral head, providing an osteoinductive and osteoconductive graft source without donor-site morbidity. In the revision setting, however, the humeral head is often no longer available, and autograft harvest is associated with donor-site morbidity, prolonged operative time, and post-operative pain. Allograft eliminates these donor-site complications and carries a low risk of disease transmission and immunologic rejection but consists of non-vital bone with a higher risk of non-union, and importantly, in the setting of infection, carries a substantial risk of acting as a sequestrum. As an alternative to bone grafting, augmented baseplates have emerged as an option for managing glenoid bone loss and deformity, with comparable complication and revision rates reported in literature.^24^

In current practice, decision-making and successful treatment in infected cases of rTSA remain a challenge, among other things due to rising patient age, increasing comorbidity, and heterogeneity of germs, including the presence of multiresistant organisms.^1^^,^^3^^,^^12^^,^^33^^,^^36^^,^^37^ While evidence suggests that single-stage revision may achieve comparable or superior rates of infection eradication with lower overall complication rates,^13^ treatment commonly involves a two-stage approach with rTSA removal and implementation of antibiotic-loaded cement spacers for a period of several months prior to rTSA reimplantation. However, the explantation of a well-fixed baseplate inevitably results in significant (iatrogenic) bone loss, frequently enlarging the glenoid defect to a degree that necessitates a biological rTSA with structural bone grafting, or even a custom-made prosthesis, each carrying their own considerable risks, particularly in the high-risk setting of revision after PJI.

A possible emerging alternative to address glenoid bone defects in this context is the use of an injectable synthetic bone graft substitute, Cerament G (Bonesupport AB, Lund, Sweden), composed of 60% calcium sulfate and 40% hydroxyapatite, delivering 17.5 mg gentamicin per mL of paste.^4^^,^^16^^,^^34^ The dual-phase composition is designed so that the more rapidly resorbing calcium sulfate component allows early local antibiotic release, while the slower-resorbing hydroxyapatite scaffold purportedly supports sustained osteoconduction and bone ingrowth as the material gradually converts to host bone, typically over 6-12 months.^4^^,^^11^^,^^20^^,^^34^ Locally, gentamicin concentrations reach around 100-fold above the minimum inhibitory concentration for susceptible pathogens such as *Staphylococcus aureus* and *Pseudomonas aeruginosa*, while systemic serum levels remain well below the toxicity threshold.^4^^,^^28^ Whether the presence of gentamicin affects the rate or quality of bone conversion remains incompletely studied; a rat osteomyelitis model reported improved bone healing following the addition of gentamicin to the calcium sulfate/hydroxyapatite matrix, suggesting no detrimental, and possibly a beneficial, effect on osteoconduction in the infected setting, although dedicated mechanistic studies are lacking.^9^ By filling the glenoid defect at the time of rTSA explantation, this approach simultaneously treats local infection while restoring and maximizing bone stock, potentially reducing the glenoid defect grade prior to rTSA reimplantation. This may reduce the extent of residual bone defect, potentially obviating the need for biological rTSA or custom implants in selected patients. Furthermore, 3-dimensional computed tomography (CT) reconstruction during the interval period enables precise patient-specific pre-operative planning of optimal baseplate positioning, including inclination, version, and screw trajectory, while also informing the decision of whether a standard revision baseplate will suffice or whether biological rTSA with structural grafting is required, and if so, in what dimensions.

The purpose of this study is to present a case of rTSA implantation following a proximal humerus fracture (PHF), complicated by PJI, successfully managed with a novel revision strategy combining an antibiotic-eluting bone graft substitute and 3-dimensional CT-guided planning for reimplantation. This strategy was based on the proposed osteoconductive and antimicrobial properties of Cerament G.

### Case presentation

A 58-year-old woman presented to the emergency department in September 2023 following a low-energy fall after tripping over a tile. She landed directly on her right shoulder, which immediately caused a lot of pain. Her medical history included type 2 diabetes mellitus, a prior deep venous thrombosis of the right leg with pulmonary embolism, and a cephalosporin allergy. Physical examination revealed a painful right shoulder in an abnormal position, without evident skin dimpling. Imaging, including plain radiographs and CT, confirmed a complex PHF with anteroinferior dislocation of the articular segment (Association for the Study of Internal Fixation (AO) classification 11C) (Fig. 1).

**Figure 1:**
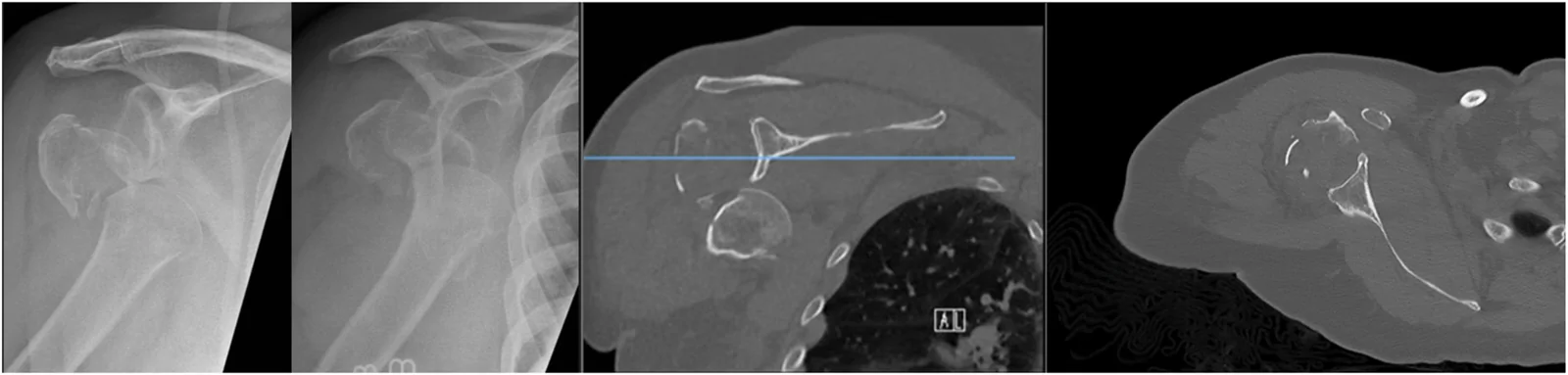
Imaging confirming complex proximal humerus fracture with anteroinferior dislocation of the articular segment of the right shoulder, Association for the Study of Internal Fixation classification 11C.

### Surgical course

On the day of presentation, surgical intervention was commenced with the intention of open reduction and internal fixation. However, upon attempted reduction of the humeral head, significant bleeding from the posterior circumflex humeral artery was encountered, which could not be safely controlled. Therefore, an osteotomy of the coracoid process was performed to improve access, and the procedure was limited to repositioning of the PHF without fixation.

Given prior unsuccessful surgery, the fracture complexity and degree of fracture dislocation, and the patient's age and bone quality, rTSA was performed 3 weeks after presentation using an Affinis Invhys Ltd, Bettlach, Switzerland) with a titanium baseplate and a vitamin E-infused, highly cross-linked polyethylene glenosphere (Vitamys). The post-operative course was initially satisfactory, with no clinical or radiographic signs of infection (Fig. 2).

**Figure 2:**
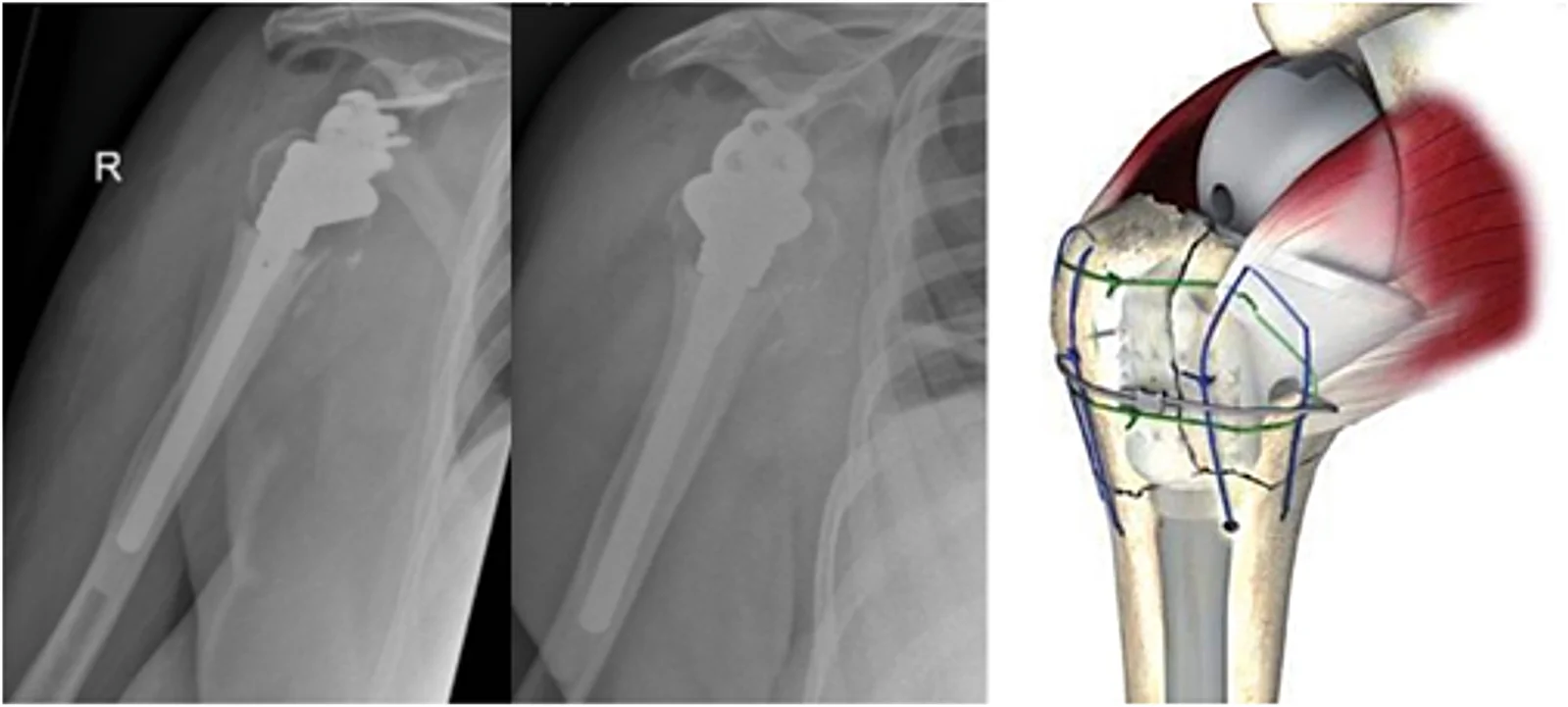
Post-operative imaging after reverse shoulder arthroplasty surgery.

Approximately 7 months post-operatively (May 2024), the patient reported progressive shoulder pain and declining function. Single-photon emission computed tomography and leukocyte scintigraphy demonstrated signs of glenoid baseplate implant loosening, with inconclusive but suspicious concurrent uptake for PJI (Fig. 3). The diagnostic accuracy of nuclear imaging for PJI is highly dependent on pretest probability^17^^,^^31^; in this patient, the combination of a clearly loosened baseplate on conventional imaging, significant clinical symptoms, and suspicious nuclear imaging findings was together considered sufficient to warrant surgical exploration.

**Figure 3:**
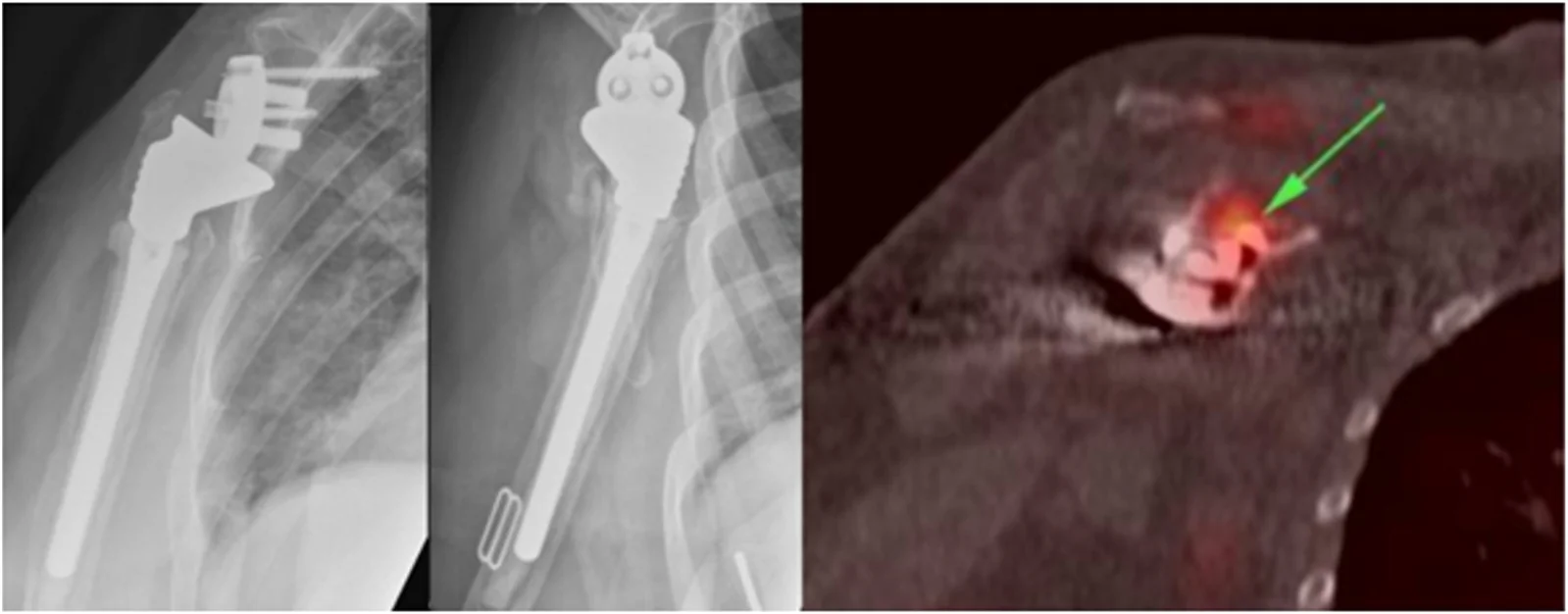
Imaging showing signs of glenoid baseplate implant loosening and concurrent findings suspicious for prosthetic joint infection.

In July 2024, the rTSA components were surgically removed, and 6 intraoperative tissue cultures were obtained, all yielding growth of *Staphylococcus epidermidis*. Antibiotic treatment was administered according to the Dutch SWAB guideline for bone and joint prosthetic infections.^32^ It consisted of 2 weeks of intravenous vancomycin, followed by oral trimethoprim-sulfamethoxazole combined with rifampicin until reimplantation. At the time of explantation, a type 2 Göhlke-Seebauer C2 glenoid bone defect was identified following baseplate removal. An antibiotic-loaded cement spacer was placed, and antibiotic-eluting bone graft substitute Cerament G was applied to fill the glenoid defect (Fig. 4).

**Figure 4:**
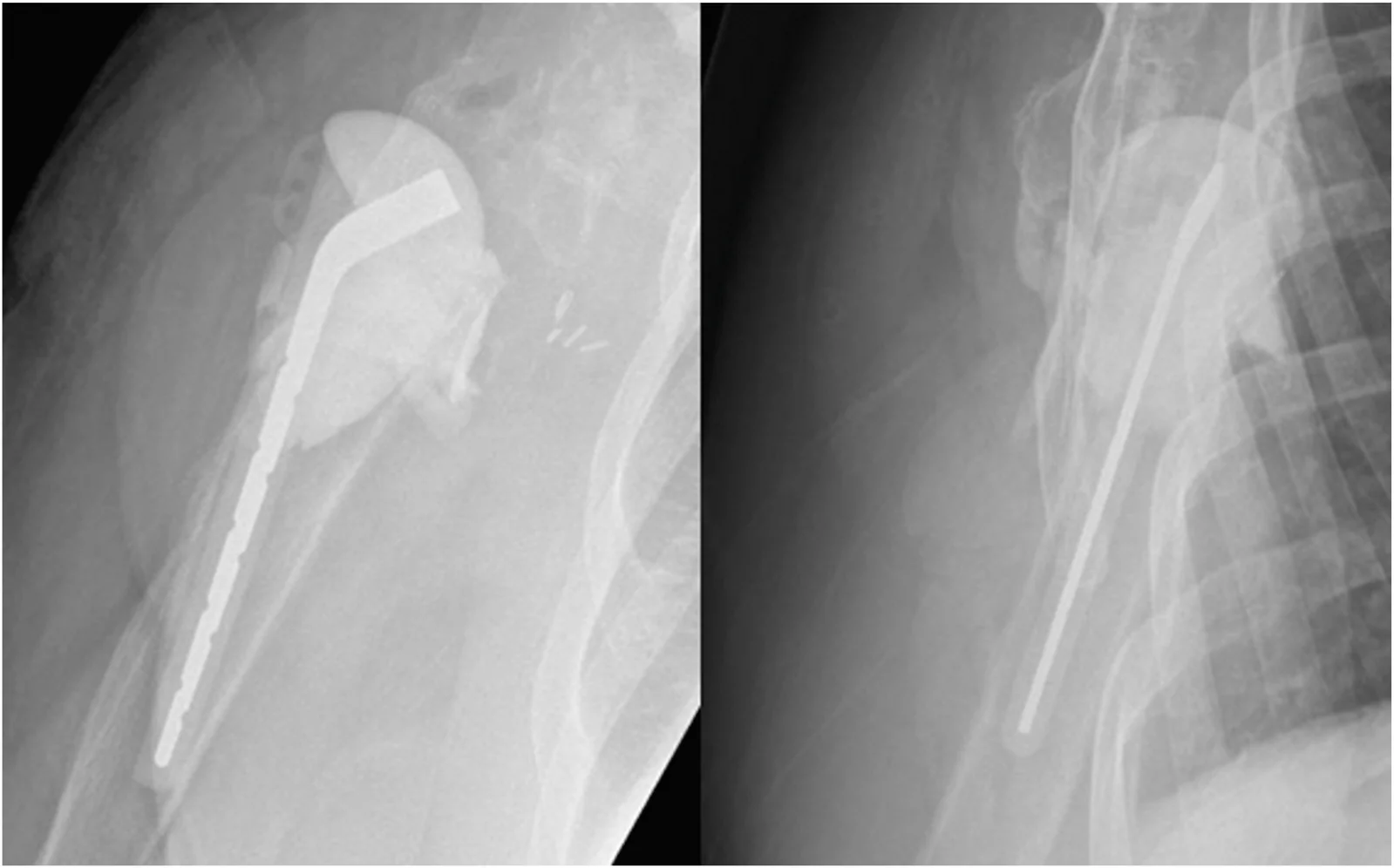
Imaging showing an antibiotic-loaded cement spacer with antibiotic-eluting bone graft substitute Cerament G to fill the glenoid defect.

An interval planning CT with 3-dimensional reconstruction was performed 7 weeks after surgical removal of the rTSA components to assess residual bone stock, guide optimal baseplate positioning, including inclination, version, and screw trajectory, and determine whether structural bone grafting would be required for reimplantation (Fig. 5).

**Figure 5:**
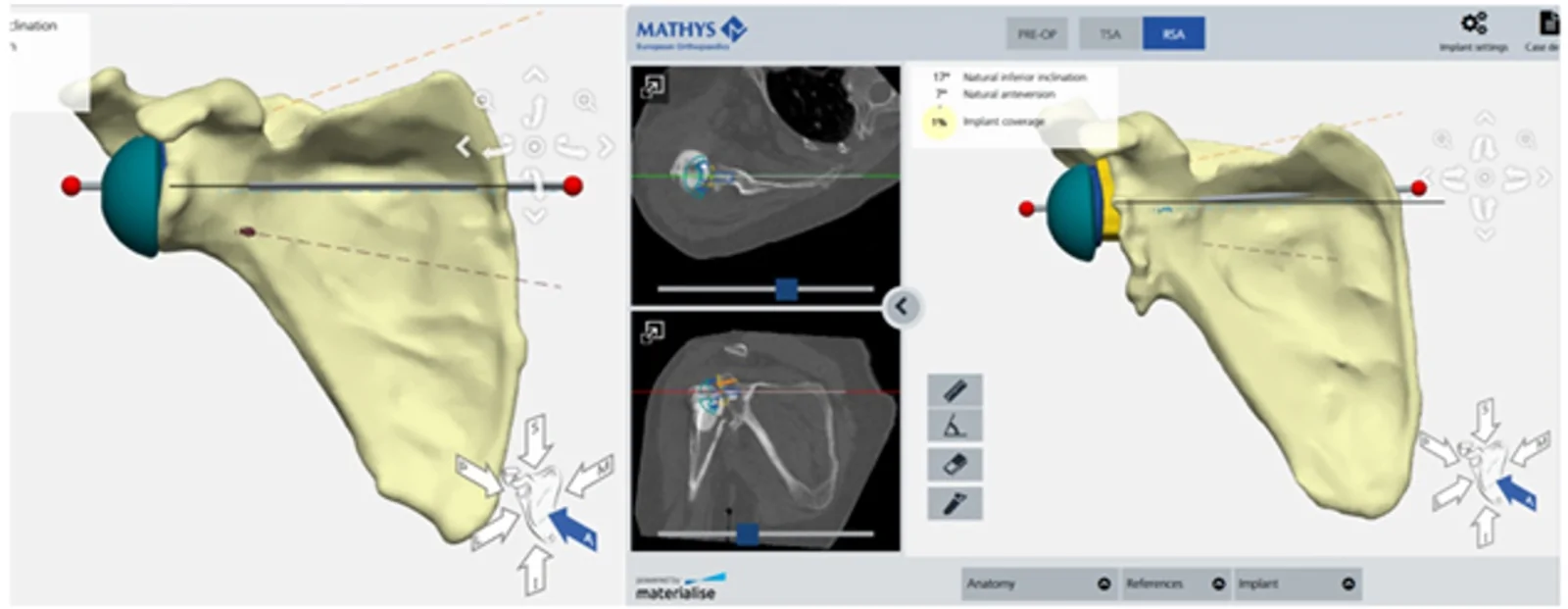
Interval planning computed tomography with three-dimensional reconstruction.

In September 2024, one week after performing the CT with 3-dimensional reconstruction, the spacer was removed, and repeat intraoperative cultures returned no growth. rTSA reimplantation was performed using an Affinis Inverse Fracture prosthesis with a titanium baseplate and Vitamys polyethylene glenosphere in accordance with the pre-operative 3-dimensional CT-based plan. Perioperative antibiotic prophylaxis consisted of 24 hours of clindamycin due to the patient's cephalosporin allergy. Notably, adequate bone stock had been restored by the Cerament G, allowing optimal baseplate placement without the need for additional bone grafting or biological rTSA (Fig. 6). The post-operative course was uneventful, and culture-guided trimethoprim-sulfamethoxazole antibiotic therapy was continued for the final 4 weeks following reimplantation, completing a total antibiotic treatment duration of 12 weeks.

**Figure 6:**
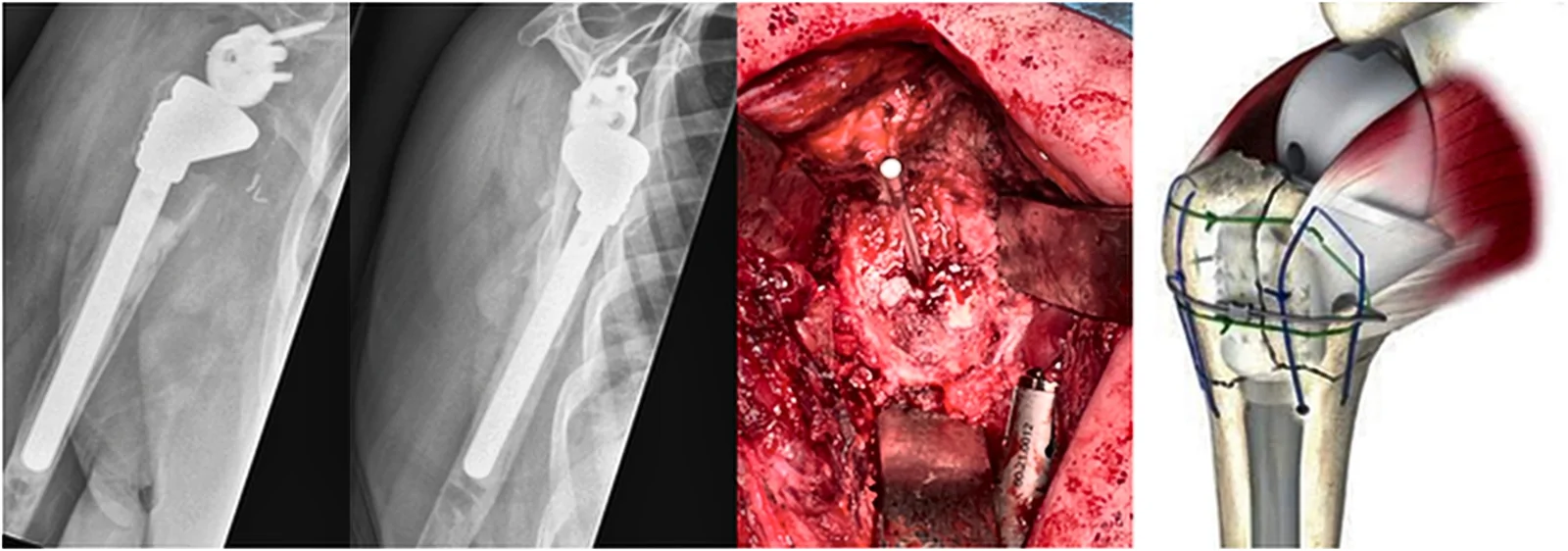
Intraoperative and radiology images showing adequate glenoid bone stock and reverse shoulder arthroplasty reimplantation without the need for additional bone grafting/biological rTSA.

At a follow-up of 4 and a half months (January 2025) after reimplantation (September 2024), the patient reported no pain and showed no clinical or radiological signs of infection (Fig. 7). Functional assessment demonstrated active range of motion of 90° of flexion, 90° of abduction, 10° of external rotation, and 10° of internal rotation.

**Figure 7:**
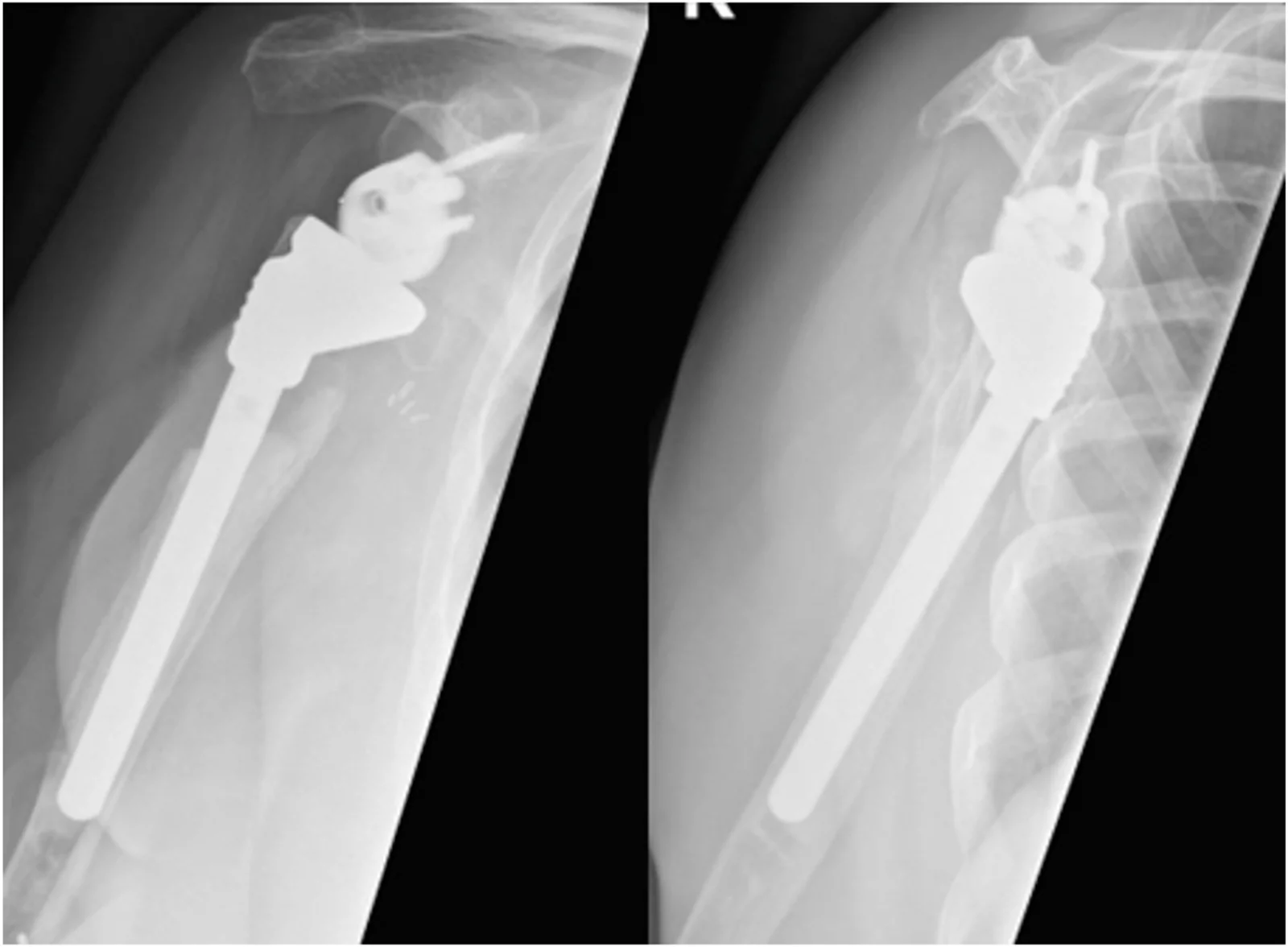
Post-operative imaging 4 and a half months after reverse shoulder arthroplasty reimplantation surgery.

At a follow-up of approximately 1 and a half years (March 2026), the patient remained pain-free without any clinical or radiological signs of infection (Fig. 8). Functional assessment demonstrated active range of motion of 110° of flexion, 95° of abduction, 10° of external rotation, and 10° of internal rotation, representing a modest improvement compared to the previous assessment.

**Figure 8:**
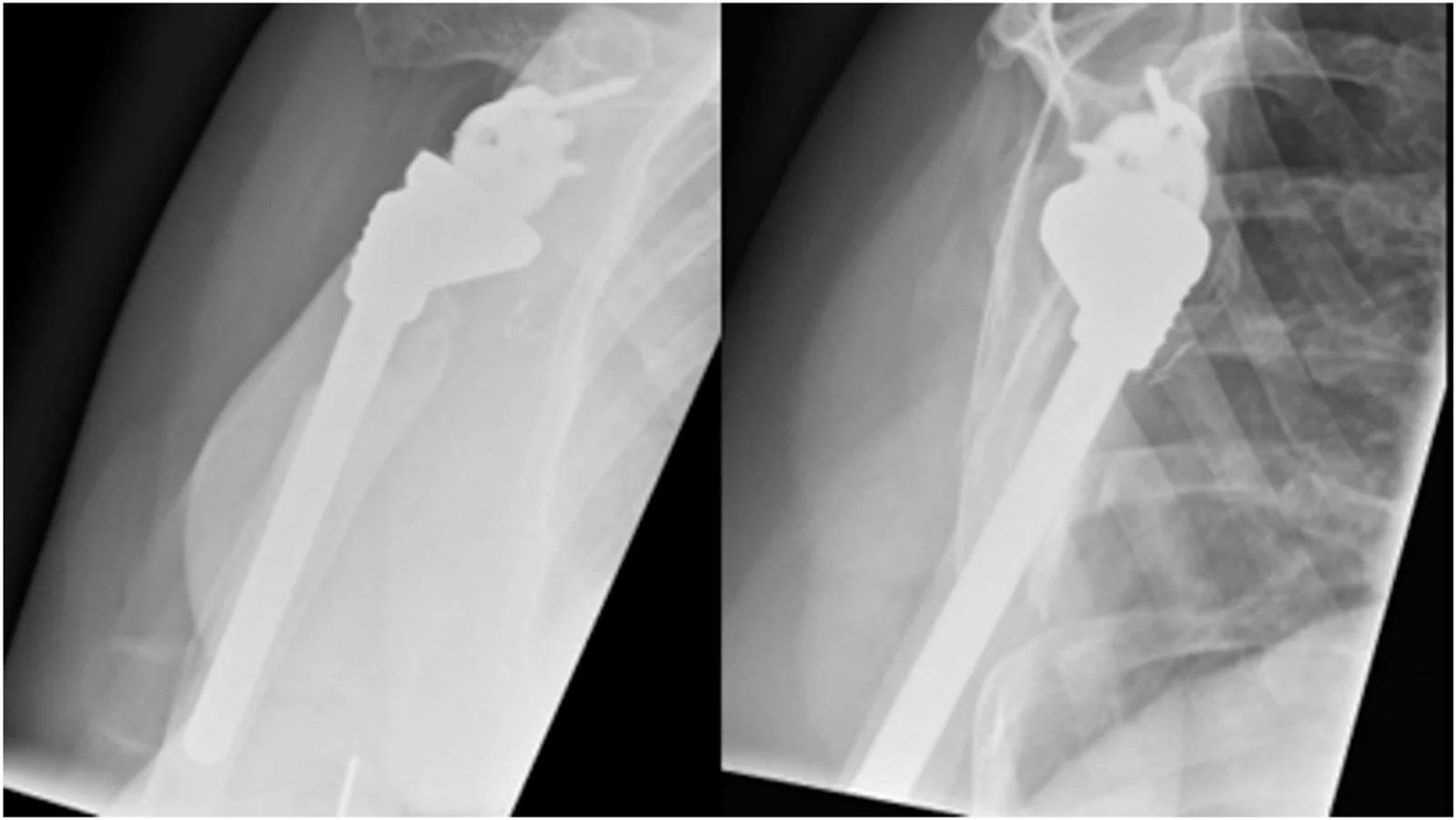
Post-operative imaging 1 and a half years after reverse shoulder arthroplasty reimplantation surgery.

## Discussion

Cerament G is a calcium sulfate/hydroxyapatite composite reported to combine local antibiotic elution with osteoconductive and purposedly osteoinductive, properties.^6^^,^^9^^,^^10^^,^^14^^,^^15^^,^^20^^,^^30^ This approach theoretically addresses 2 key challenges in glenoid revision surgery in the management of PJI following rTSA, eradication of local infection, and restoration of bone stock within a single biomaterial application. Whether progressive remodeling of the glenoid architecture during the interval period can meaningfully reduce defect severity prior to reimplantation and potentially reduce the need for biological bone grafting in Göhlke type 1 and type 2 defects, or selected type 3 cases, and Seebauer C1 to 3 defects, remains to be established in larger prospective studies. This could potentially represent a meaningful shift in the reconstructive strategy for this challenging patient population.

To our knowledge, no studies directly comparing Cerament G and conventional approaches, typically combining antibiotic-loaded cement spacers with biological glenoid reconstruction, in revision rTSA have been published, representing an important gap in current literature. Antibiotic-loaded cement spacers (polymethyl methacrylate) remain the established standard of care for PJI treatment, with a well-documented track record of infection control rates exceeding 85% in 2-stage exchange arthroplasty.^2^^,^^8^^,^^12^ However, polymethyl methacrylate is a non-resorbable, inert material that lacks bioactivity and does not support osseointegration or bone regeneration. Moreover, the exothermic polymerization of bone cement may theoretically induce thermal injury to surrounding bone and tissue, although the clinical significance of this effect in practice remains uncertain. Consequently, while conventional cement spacers effectively occupy the dead space during the interval period, they do not contribute to restoration of bone stock, meaning that upon spacer removal, the bone defect remains unchanged in size or may have further progressed. In contrast, antibiotic-eluting bone graft substitute Cerament G is advocated to fulfill the same antibiotic delivery function while also acting as a resorbable scaffold that promotes new bone formation throughout the interval period, raising the intriguing prospect of single-stage revision protocols in carefully selected patients.

The current study is the first to describe the use of Cerament G in glenoid bone defects following revision rTSA. Supporting evidence from analogous settings in other anatomical regions provides a meaningful basis for its application here. In the context of hip revision arthroplasty, Khakzad et al^22^ reported the safety and feasibility of single-stage cementless hip exchange arthroplasty using Cerament G for the management of PJI and associated femoral and acetabular bone defects, demonstrating significant improvements in functional outcomes and quality of life with a favorable safety profile. Similarly, Stravinskas et al^34^ investigated local Cerament G application in patients undergoing trochanteric femoral fracture fixation or uncemented hip revision, finding no infection at 1-year follow-up, radiological evidence of bone remodeling, and new bone formation in all cases, with histology confirming viable and mature bone at the implantation site. The broader evidence base has been summarized in a scoping review by Bove et al^6^ which included 22 studies and concluded that it offers a versatile and effective solution for bone reconstruction across multiple orthopedic disciplines, through its clinical performance, ease of use, and antimicrobial capabilities supporting integration into routine practice. The strongest evidence for infection eradication comes from the field of chronic osteomyelitis. McNally et al^27^, ^28^, ^29^ reported a 94% infection eradication rate in a prospective series of 100 patients with a mean follow-up of 6 years following single-stage treatment with Cerament G, alongside an infectious persistence rate of approximately 4-5%. Ferguson et al^10^ further corroborated these findings, reporting a 96% eradication following a single procedure, with bone void filling observed in 74% of cases and continued bone formation beyond two years in nearly two-thirds of patients, and histology confirming active biomaterial remodeling.

Unlike conventional bone grafts, which must be removed or reamed away at the time of revision, Cerament G is gradually converted into host bone and can be left in situ, avoiding additional iatrogenic bone loss at the time of reimplantation. Notably, Freischmidt et al^14^ demonstrated promising results for implants (plates, intramedullary nails, or prostheses) coated with Cerament G, raising the question of whether its application could extend beyond treatment to prophylaxis, potentially reducing the risk of PJI in selected high-risk patients at the time of primary or revision rTSA.

Nevertheless, it is important to acknowledge that the efficacy of Cerament G is influenced by the local biological environment at the implantation site. Kavarthapu et al^21^ reported a non-union rate of 24% when Cerament G was used in infected Charcot foot bones, raising the possibility that the healing response to a biocomposite bone graft substitute may be attenuated in the setting of neuropathic or severely compromised tissue. Calcaneal osteomyelitis was additionally associated with a risk of recurrent infection. Likewise, Niemann et al^30^ reported favorable functional outcomes and patient-reported outcome measures in chronic osteomyelitis, while noting a higher rate of local wound complications and osteomyelitis persistence compared to previously published data. Freischmidt et al^15^ further investigated Cerament G in an animal model of infected and non-infected non-unions. Their data show osteoinductive properties but no added benefit in infected non-unions in terms of osteoconduction and mechanical bone stability, particularly in segmental defects. Therefore, only a prophylactic, rather than eradicative, effect against infection could be verified in the microbiological analysis, with osteoconduction observed exclusively in the non-infected setting. This is a clinically relevant distinction, as performance seems most effective when the infectious burden has already been reduced. In the present case, application followed adequate surgical débridement and was accompanied by systemic antibiotic therapy, which likely contributed to the favorable outcome. These findings serve as a reminder that, while Cerament G offers a promising multifunctional solution, careful patient selection, thorough débridement, and appropriate systemic antibiotic coverage remain essential points of consideration.

Future research should explore whether the bone stock restoration achieved with Cerament G is sufficient to enable single-stage revision protocols, which would represent a significant step forward in reducing treatment burden for affected patients. Additionally, the potential applications of Cerament G in arthroplasty warrant systematic investigation as it may extend beyond the shoulder given the universal challenge of periprosthetic bone loss and PJI across joint replacements. Furthermore, the concept of prophylactic application, for instance, by implant coating in high-risk patients, such as those undergoing revision surgery, immunocompromised patients, or patients with prior infection, represents a clinically relevant perspective.

Several limitations of this case report warrant consideration. As a single case, it is inherently limited in its capacity to establish generalizability or draw definitive conclusions regarding the efficacy of Cerament G in glenoid bone defects following revision rTSA. Larger prospective studies and comparative cohorts are needed to validate the findings presented and to determine in which defect types this approach is most reliably effective. Furthermore, the economic implications also merit attention, as Cerament G carries a considerably higher material cost compared to conventional antibiotic-loaded cement spacers or allograft. While this cost may potentially be offset by a reduction in surgical complexity at reimplantation, for instance by avoiding biological rTSA or custom implants, formal cost-effectiveness analyses are currently lacking. Additionally, as is inherent to retrospective reporting, some intraoperative and post-operative images were of limited resolution; these images were intended to illustrate the clinical course rather than to serve as the primary basis for defect assessment, which relied on intraoperative measurement and 3-dimensional CT reconstruction. Finally, although the follow-up period of 1 and a half years following reimplantation is encouraging, it remains insufficient to draw conclusions regarding long-term implant survival, durability of bone regeneration, and infection recurrence. Extended follow-up will be essential to determine observed outcomes over time. Further biomechanical and population studies are needed to validate the hypotheses raised by this report.

## Conclusion

This case demonstrates that the antibiotic-eluting bone graft substitute Cerament G is a promising adjunct in 2-stage revision rTSA for PJI, addressing both infection eradication and glenoid bone stock restoration. It potentially reduces defect severity prior to reimplantation, as illustrated by the favorable short-term outcome in this case. This is the first report describing its application in this context. Larger prospective studies are warranted to validate these findings, to explore its efficacy across the spectrum of glenoid bone loss severity, and to assess its potential role in prophylactic use and single-stage revision protocols.

## Disclaimers:

Funding: No funding was disclosed by the authors.

Conflicts of interest: The authors, their immediate families, and any research foundations with which they are affiliated have not received any financial payments or other benefits from any commercial entity related to the subject of this article.

Patient consent: Obtained.

## Declaration of generative AI and AI-assisted technologies in the writing process

During the preparation of this work, the author(s) used generative AI (ChatGPT, OpenAI, Claude.ai) in order to assist with readability, grammar, and concise rephrasing. It was not used to generate or interpret data, nor to produce figures or statistical results. After using this tool/service, the author(s) reviewed, validated, and edited the content as needed and take(s) full responsibility for the content of the published article. Graphical abstract was designed by the authors and illustrated using BioRender.com.
